# Clinical translation of gold nanoparticles

**DOI:** 10.1007/s13346-022-01232-4

**Published:** 2022-08-31

**Authors:** Rui Zhang, Fabian Kiessling, Twan Lammers, Roger M. Pallares

**Affiliations:** Institute for Experimental Molecular Imaging, RWTH Aachen University Hospital, 52074 Aachen, Germany

**Keywords:** Gold nanoparticles, Gold nanoconstructs, Nanomedicine, Clinical trials, Drug delivery, Photothermal therapy

## Abstract

Gold nanoparticles display unique physicochemical features, which can be useful for therapeutic purposes. After two decades of preclinical progress, gold nanoconstructs are slowly but steadily transitioning into clinical trials. Although initially thought to be “magic golden bullets” that could be used to treat a wide range of diseases, current consensus has moved toward a more realistic approach, where gold nanoformulations are being investigated to treat specific disorders. These therapeutic applications are dictated by the pharmacokinetics and biodistribution profiles of gold nanoparticles. Here, we analyze the current clinical landscape of therapeutic gold nanoconstructs, discuss the shared characteristics that allowed for their transition from bench to bedside, and examine existing hurdles that need to be overcome before they can be approved for clinical use.

**Graphical Abstract:**

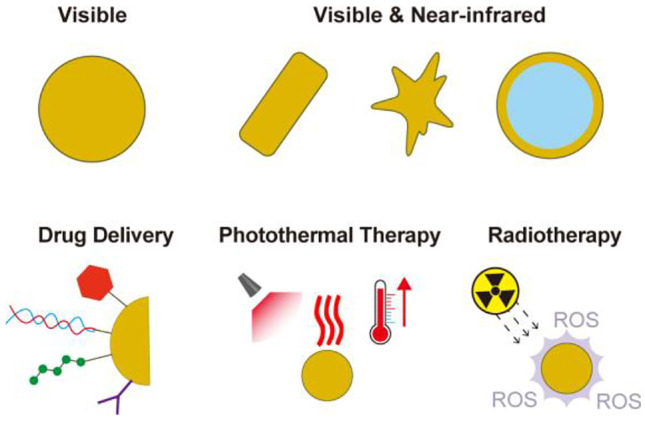

## Introduction

**Fig. 1:**
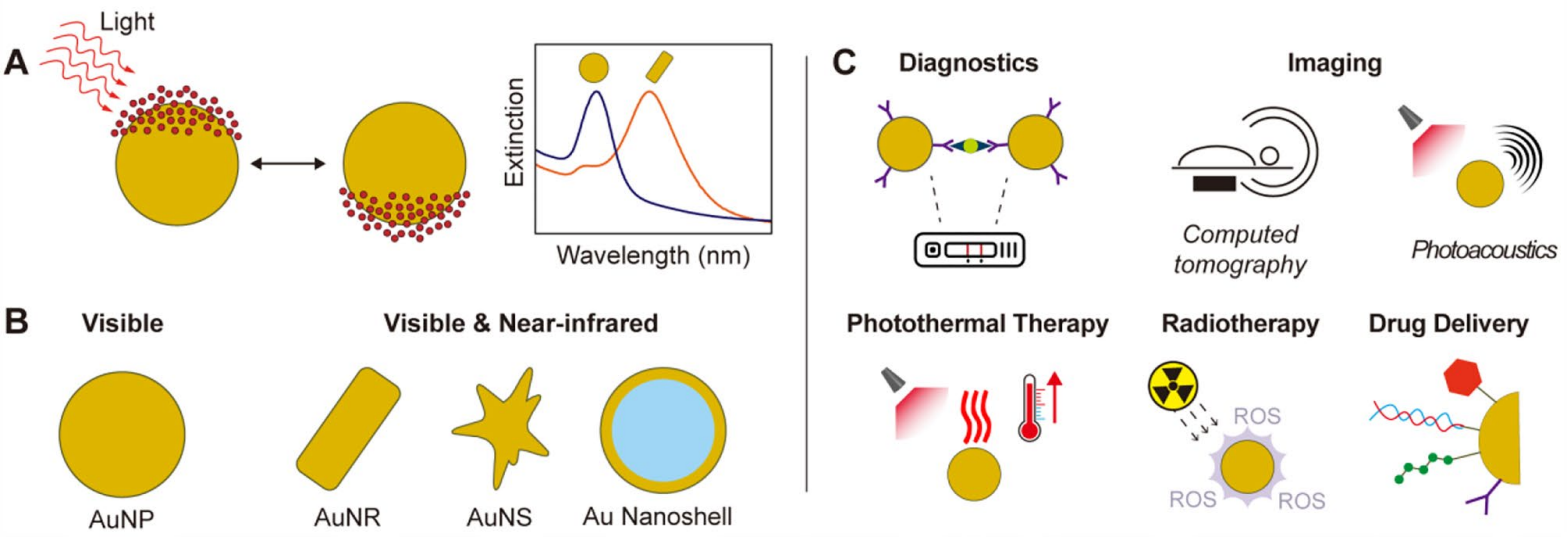
Fundamentals of gold nanoparticles. **A** Schematic representation of localized surface plasmon resonance (i.e., collective oscillation of the conduction band electrons of gold nanoparticles, when interacting with an electromagnetic radiation), and the resulting extinction spectrum. **B** Gold nanorods (AuNR), gold nanostars (AuNS), and gold nanoshells (Au nanoshell) display distinct optical properties compared to spherical nanoparticles (AuNP). **C** Different biomedical applications of gold nanoconstructs. ROS stand for reactive oxygen species

In the last two decades, gold nanoparticles (AuNP) have attracted extensive interest [1]. AuNP display unique optoelectronic properties [2], which originate from their localized surface plasmon resonances (i.e., collective oscillation of their conduction band electrons when interacting with light of specific wavelengths, Fig. 1A). These optoelectronic properties can be fine-tuned through colloidal chemistry, via engineering nanocrystal size and shape [3]. For example, anisotropic AuNP, such as high aspect ratio gold nanorods, gold nanostars, and thin gold nanoshells, display strong extinction coefficients in the near-infrared region (Fig. 1B) [4–7], where light shows deeper tissue penetration. As such, AuNP have expanded the medical applications of gold, which historically had been limited to the use of gold salts for the treatment of rheumatoid arthritis [8]. In (pre)clinical settings, modern use of AuNP (Fig. 1C) includes point-of-care diagnostics (lateral flow assays, such as pregnancy tests and rapid COVID-19 tests, and surface enhanced Raman spectroscopy [9, 10]), in vivo imaging (photoacoustics and computed tomography [11, 12]), and therapeutics (photothermal therapy, radiotherapy, catalytic therapy, and drug delivery [13–15]). As an example of the latter, AuNP absorbing light in the near-infrared region have been used for thermal ablation of solid tumors, with AuNP converting the energy of the photons into local heat that triggers apoptosis and necrosis of cancerous cells [16]. Furthermore, AuNP profit from ease of synthesis as well as surface functionalization, allowing the conjugation of therapeutic, targeting, and stabilizing agents on their surface [17], and thereby prolonging blood circulation (compared to small molecules) and promoting accumulation at pathological regions, including tumors and sites of inflammation [18, 19]. As a result, AuNP have been used as drug carriers for chemotherapeutics, immunomodulatory agents, and nucleic acids (e.g., siRNA and aptamers), among others.

## Clinical landscape of therapeutic gold nanoparticles

Despite the large amount of literature reporting new and exotic therapeutic formulations based on AuNP, few gold nanoconstructs have made it to clinical trials. This inconsistency between preclinical and clinical studies is caused by most research being materials science-driven, where the aim is to develop multifunctional NP with new properties, rather than focusing on promoting characteristics needed in the clinic. For example, both inorganic and organic NP that have reached the clinic tend to be simple and composed of only a few components, making their in vivo behavior easier to predict and their production easier to scale up [20]. Such simple nanoconstructs, however, are relatively rare in basic science studies. Another factor that has been limiting AuNP translation is their incomplete excretion in vivo, as a significant fraction of administered AuNP tend to accumulate in certain organs, such as liver and spleen [21]. Although AuNP may not exhibit acute toxicity, there are concerns regarding long-term side effects. Considering the above, we here summarize the current clinical landscape of therapeutic AuNP. By analyzing the characteristics of gold nanoconstructs that are explored in the clinic (Table 1), we discuss how AuNP can provide real value for patient treatment.

**Table 1:** Therapeutic gold nanoconstructs in clinical trials

**Name**	**NP type**	**Application**	**Clinical trial**
CYT-6091	PEGylated 27-nm AuNP functionalized with TNF	Anti-tumor therapy via immune response regulation	NCT00356980, Phase 1, Completed (2006 – 2009) NCT00436410, Phase 0, Completed (2006 – 2009)
C19-A3 AuNP	Human proinsulin peptide (C19-A3) conjugated to ultrasmall AuNP (< 5 nm)	Treatment of the autoimmune disorder type 1 diabetes	NCT02837094, Phase I, Active (2016 –)
EMX-001 (naNO-DENGUE)	T-cell priming cocktails of peptides of dengue virus conjugated on the surface of AuNP	Vaccines against dengue fever	NCT04935801, Phase I, Active (2021 –)
naNO-COVID	T-cell priming cocktails of peptides of coronavirus conjugated on the surface of AuNP	Vaccines against SARS-CoV-2	NCT05113862, Phase I, Active (2022 –)
NU-0129	siRNA and thiolated PEG arranged on the surface of 13-nm AuNP	Treatment of glioblastoma	NCT03020017, Phase 0, Completed (2017 – 2020)
AuroShell	PEGylated 120-nm silica core and 12 to 15-nm gold shell	Photothermal ablation of different types of cancer	NCT00848042, Completed (2008 – 2014) NCT01679470, Terminated (2012 – 2014) NCT02680535, Completed (2016 – 2020) NCT04240639, Recruiting (2020 –)
Gold nanoshells	60 to 70-nm silica core and 15 to 40-nm gold shell	Photothermal ablation of atherosclerotic plaques	NCT01270139, Completed (2007 – 2016) NCT01436123, Terminated (2010 – 2012)
CNM-Au8	13-nm AuNP in drinkable bicarbonate solution	Treatment of several neurodegenerative diseases	NCT02755870, Phase I, Completed (2015 – 2016) NCT03536559, Phase II, Active (2018 –) NCT03815916, Phase II, Completed (2019 – 2021) NCT03993171, Phase II, Recruiting (2019 –) NCT04081714, Available (2019 –) NCT04098406, Phase II, Completed (2019 – 2021) NCT04414345, Phase II/III, Active (2020 –) NCT04626921, Phase II/III, Active (2020 –) NCT05299658, Phase II, Active (2021 –)

**Fig. 2:**
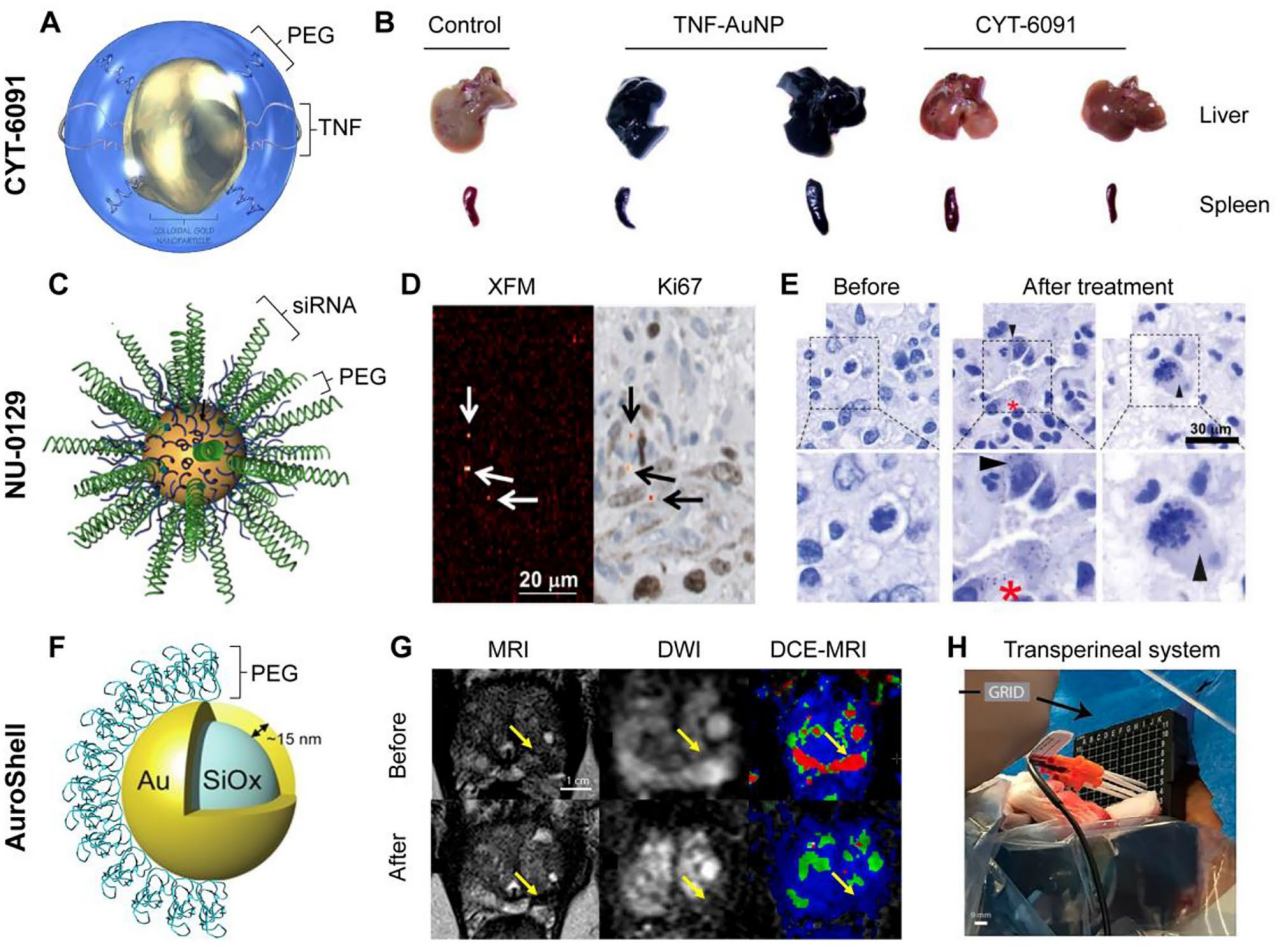
Representative therapeutic gold nanoconstructs in clinical trials. **A** Schematic representation of CYT-6091. **B** Comparison of ex vivo organ accumulation of non-PEGylated (TNF-AuNP) and PEGylated CYT-6091. Adapted with permission of ref 22. Copyright 2012 CytImmune. **C** Schematic representation of NU-0129. **D** Au elemental map of glioblastoma tumor sample and matching Ki67 staining after NU-0129 treatment. Arrows highlight gold accumulation regions within perivascular Ki67-positive cancerous cells. **E** Silver staining of glioblastoma tumors before and after NU-0129 treatment. Adapted with permission of ref 36. Copyright 2020 American Association for the Advancement of Science. **F** Schematic representation of AuroShell. Adapted with permission of ref 38. Copyright 2015 Elsevier. **G** Pre- and post-treatment images of patient with focal prostate cancer treated with AuroShell and photoablation. Imaging techniques: t2-weighted magnetic resonance imaging (MRI), diffusion-weighted imaging (DWI) and dynamic contrast-enhanced magnetic resonance (DCE-MRI) imaging. Arrows highlight tumoral region. **H** Picture of lasers introducers in thermocouple through transperineal grid. Adapted with permission of ref 40. Copyright 2019 National Academy of Science

CYT-6091 is a PEGylated-AuNP functionalized with recombinant human tumor necrosis factor alpha (TNF) (Fig. 2A), a pro-inflammatory cytokine released by leukocytes upon activation [22]. TNF has been widely explored in preclinical and clinical cancer research because of its anti-tumor effect via immune response regulation [23]. However, since TNF receptors are widely expressed on many cells in many tissues, generalized TNF activation can induce strong systemic inflammation and toxicity, potentially resulting in septic shock and patient death. Local delivery of TNF has the potential of conveying proper therapeutic efficiency while avoiding systemic side effects. AuNP can be directly conjugated with TNF via thiol residues without the need for using further chemicals. After PEGylation, the circulation time of TNF-AuNP is significantly increased [24], as the particle uptake and clearance by the reticuloendothelial system is reduced (Fig. 2B). Moreover, the higher tumor accumulation of TNF-AuNP compared to free TNF and non-PEGylated TNF-AuNP has led to better therapeutic outcomes in animal models [24]. The first clinical trial (NCT00356980), which was initiated in 2006 was a phase I dose escalation study in which 27-nm AuNP conjugated with TNF and thiolated PEG were intravenously injected to patients with advanced solid tumors [25]. Each patient received one cycle consisting of two injections, administered 14 days apart. The therapeutic doses ranged from 50 to 600 μg/m^2^, which were all well tolerated. Administration of TNF as a nanoformulation allowed to exceed the maximum tolerated dose of TNF (i.e., compared to parental free protein treatment) by threefold, without showing any significant side effect. Twenty-four hours post-administration, electron microscopy confirmed the presence of AuNP in tumor tissue biopsies. A phase 0 clinical trial (NCT00436410) was started short after, also in 2006, where the tissue distribution and toxicity of intravenously injected CYT-6091 was studied in patients undergoing surgery for primary or metastatic cancer. In July 2020, it was announced that Cytimmune (the biotechnology company behind the development of CYT-6091) had signed a clinical trial agreement with the National Cancer Institute to carry on a phase II clinical trial of CYT-6091. The starting date for this study has yet to be defined.

Human proinsulin peptide (C19-A3) conjugated to ultrasmall AuNP (C19-A3 AuNP) has been tested for the treatment of the autoimmune disorder type 1 diabetes, in which pathogenic T cell activation damages insulin-secreting β cells. Downregulating T cell responses with antigen-specific peptides, such as C19-A3 [26], offers the opportunity to restore immune homeostasis and prevent tissue damage in a targeted manner, without systemic immune suppression. While the administration of the antigen alone (and also with adjuvants) has been proven to be safe, achieving sufficient therapeutic effect to reverse the disease has not been possible, because of low stability and inefficient delivery to immune cells [27]. Thus, ultrasmall AuNP (< 5 nm) were proposed as delivery carriers of C19-A3, as they can be easily functionalized and present higher uptake by antigen-presenting cells than free peptides. The safety profile of C19-A3-conjugated AuNP intradermally administered via microneedles (three doses at 4-week intervals) was explored in a phase I clinical trial, starting in 2016 [28]. The type 1 diabetes patients tolerated the C19-A3-AuNP doses well, and no signs of systemic hypersensitivity were observed. The only side effects reported were delayed skin reaction at the injection site, which gradually faded over 12 to 24 months, and local gold hypersensitivity, which was assessed by epicutaneous patch test. Moreover, there was no evidence of systemic gold retention after several weeks post-injection, but prolonged retention of AuNP in the skin was observed. The benefits of this approach (good tolerance, minimally invasive administration route, and potential therapeutic effect) may outweigh the risks (local gold retention and hypersensitivity); however, future clinical studies (phase II and III) are necessary to assess the therapeutic benefits.

AuNP have also been explored as drug delivery vehicles for vaccines against dengue fever (EMX-001, also referred as naNO-DENGUE) and SARS-CoV-2 (naNO-COVID). The vaccines are T cell priming cocktails of peptides of dengue virus or coronavirus conjugated on the surface of AuNP. Because NP predominantly accumulate in phagocytic cells [29], efficient delivery of the immunogenic cargo to antigen-presenting cells is ensured. Furthermore, AuNP protect peptides from premature proteolytic degradation and reduce the required antigen dose to the nanomolar range. EMX-001 (NCT04935801) and naNO-COVID-19 (NCT05113862) are now in phase I clinical trials (started in 2021 and 2022, respectively), where the safety of the nanoconstructs is being screened. In both studies, patients receive two AuNP doses intradermally injected in a 21-day interval. Two therapeutic doses are explored, namely 2.5 nmol peptide (12.8 μg gold) and 7.5 nmol peptide (38.3 μg gold). The immunogenicity is evaluated by the proportion of participants with CD8^+^ T cells specific to dengue and SARS-CoV-2 peptides, and the percentage of participants becoming seropositive (i.e., presenting antibodies against the dengue virus or coronavirus). According to Emergex Vaccines Holding (sponsor of both studies), the clinical trial of EMX-001 is fully recruited and ongoing, and the results are expected to be released in the summer of 2022. The naNO-COVID clinical study is currently ongoing with the first participant dosed on 10 January 2022.

NU-0129 consists of small interfering RNA (siRNA) and thiolated PEG arranged on the surface of AuNP (Fig. 2C), and it is being explored for the treatment of glioblastoma. RNA interference is a promising cancer treatment, as it allows to silence oncogenes [30]. For example, in glioblastoma, the Bcl2Like12 (Bcl2L12) oncoprotein tends to be overexpressed, leading to apoptosis inhibition [31]. Thus, siRNA can target and block the expression of Bcl2L12, disturbing the growth and propagation of tumor cells. However, the rapid degradation of siRNA in the bloodstream and its inefficient delivery to and into cancerous cells result in low therapeutic effects, requiring delivery systems [32]. The biological behavior of RNA changes when densely packed on the surface of AuNP, forming nanoconstructs known as spherical nucleic acids (SNA) [33]. The high-density architecture of the oligonucleotide shell in SNA results in RNA exhibiting significantly higher stability against nuclease degradation than free RNA [34]. Moreover, the SNA display polyvalent interactions and have been claimed to be able to cross the blood–brain barrier, enabling delivery of siRNA to glioblastoma lesions and knockdown of Bcl2L12, promoting apoptosis and tumor shrinkage [35]. A phase 0 clinic trial (NCT03020017) was completed in 2020 to assess the safety, intratumoral penetration, and oncoprotein-suppressive effect of NU-0129 administered to recurrent glioblastoma patients undergoing surgery [36]. NU-0129 was intravenously injected to patients at a siRNA dose of 0.04 mg/kg, 20 to 28 h prior to surgery. Patients tolerated NU-0129 well, and no high-grade adverse event was observed. Only two severe adverse events (e.g., hypophosphatemia and lymphopenia) were noted, which were both considered as “possibly” associated with the treatment. Importantly, the severity of both side effects diminished by the end of the evaluation period. NU-0129 exhibited prominent tumor accumulation (ranging between 4 × 10^9^ and 1.1 × 10^11^ particles/g of tumor tissue) (Fig. 2D and E), with 20% of intratumoral SNA being located inside tumor cells, which induced a reduction of tumor-associated Bcl2L12 protein expression. It is worth noting that long-term accumulation of AuNP was detected in tumors of two patients. Nevertheless, future clinical studies (phase II and III) will be necessary to assess the therapeutic efficacy of these nanoconstructs beyond the reported Bcl2L12 downregulation and AuNP tumor accumulation. To date, there is no evidence of further ongoing drug development.

AuroShell particles are a type of AuNP used for the photothermal ablation of solid tumors. These NP are made of a 120-nm silica core, covered by a thin (12–15 nm) gold shell (Fig. 2F), which yields AuNP that display a strong extinction coefficient in the near-infrared region of the optical spectrum [37, 38], where light possesses deeper tissue penetration properties. AuroShell particles are functionalized with thiolated PEG, which maximizes NP blood circulation times and promotes accumulation at tumor sites. AuroShell NP take advantage of the high photothermal conversion efficiency of gold, which concentrates the thermal ablation effect only on the vicinity of the irradiated AuNP, providing higher therapeutic selectivity [16]. In the clinic, AuroShell particles have been evaluated in several clinical trials. The results of the first clinical study were published in 2015, and reported the safety profile of AuroShell after single-dose intravenous administration in patients with prostate cancer [39]. Only two adverse events attributable to the NP administration were identified, namely an allergic reaction, which was resolved with antihistamines, and transient burning feeling in the epigastrium. Blood and urine analysis did not identify any changes caused by the AuroShell particles or the laser-irradiation through the 6-month evaluation period. Based on the hematologic results, no long-term effects of the treatment were expected. A follow-up clinical trial started in 2016 studied the therapeutic benefits of AuroShell-based photoablation of prostate cancer in 16 patients [40]. The therapeutic procedure was successfully carried out in 15 out of 16 patients, as one patient experienced epigastric pain after AuNP administration and did not undergo the laser treatment. No serious adverse events were reported, and all patients were discharged several hours after the therapy. The ablation areas were free of cancer in 60% and 87% of patients at 3 and 12 months post-treatment, respectively (Fig. 2G and H). Additionally, a case report (published in 2022) of a patient with prostate cancer treated with AuroShell reported 100% ablation of the segmented tumor, and 94% of the tumor margins [41]. Beyond prostate cancer, two pilot studies evaluated AuroShell efficacy in the treatment of patients with primary and/or metastatic lung tumors (NCT01679470) and recurrent head and neck tumors (NCT00848042). The studies were terminated and completed in 2014, respectively; however, their results have not yet been published. Although the clinical studies of AuroShell to treat prostate cancer were very promising, the outcome of an ongoing multi-institutional clinical trial with 45 patients and 12-month follow-up (NCT04240639) will provide more insight on the efficacy and potential clinical future of the therapy.

Beyond cancer treatment, gold nanoshells have also been used in the clinic to photothermally destroy atherosclerotic plaques and restore blood flow. In a first-in-man trial using gold nanoshells initiated in 2007 and completed in 2016 (NCT01270139; study known as NANOM-FIM and sponsored by the Ural State Medical University), the safety of two NP delivery strategies and subsequent photoablation therapy were evaluated [42]. In the first approach, 60 patients (nano-group) received the nanoshells loaded in stem cells that were grown on a (to be implanted) bioengineered on-artery patch. The role of the stem cells was two-fold, i.e., to act as delivery carriers and to help with tissue healing after the aggressive photoablation treatment. In the second approach, 60 patients (ferro-group) received, via infusion into the targeted coronary artery, a 1:1 mixture of stem cells and microbubbles both loaded with gold nanoshells and iron-labelled gold nanoshells. The iron allowed for magnetic targeting guidance, while the microbubbles were destroyed via ultrasound pulses, to release a fraction of the nanoshells in the area of interest. As a control group, 60 patients underwent stenting (stent-group). At 12 months post-treatment, the percent of atheroma volume (i.e., proportion of vessel wall occupied by atherosclerotic plaque) was the lowest in the nano-group (38%), compared to the ferro-group (39%) and the control-group (53%). Similarly, the nano-group also showed higher event-free survival compared to the other two groups (92% versus 82% and 80%, respectively). The 60-month clinical outcomes of the patients, published in 2017, corroborated the early observations that the nano-group showed the lowest mortality and the least adverse cardiovascular events [43]. Despite these promising results, additional studies with a larger cohort of patients and the involvement of other institutions are required to provide a better clinical context to these nanoformulations and advanced therapeutic interventions.

Lastly, 13-nm AuNP (CNM-Au8) in drinkable bicarbonate solutions are being explored for the treatment of several neurodegenerative disorders, including amyotrophic lateral sclerosis (ALS), Parkinson’s disease, and multiple sclerosis. The AuNP are clean-surfaced and faceted gold crystals, which show high catalytic properties. Particularly, CNM-Au8 are used to catalyze oxidation–reduction reactions to support different intracellular biological processes [44]. For example, AuNP can catalyze the oxidation of NADH to NAD^+^ [45], a fundamental cofactor for the synthesis of ATP. Hence, CNM-Au8 are expected to restore the deficits in cellular energy and to decrease the oxidative stress associated with multiple neuropathologies. Despite a preclinical study demonstrated the delivery of CNM-Au8 to the spinal cord and brain of canines after oral administration [44], there are still some concerns regarding the preferentially accumulation of bare AuNP in highly perfused organs [46] and their potential long-term toxicological effects. Nevertheless, the safety profile of CNM-Au8 (orally administered) was explored in a placebo-controlled phase I clinical trial completed in 2016 (NCT02755870). Although the results have not been published yet, Clene Nanomedicine (the company behind the development of CNM-Au8) announced in a company report that only mild and neural/gastrointestinal drug-related adverse events were observed, which supported further drug development. In July 2019, the US Food and Drug Administration granted CNM-Au8 the orphan drug designation, which promotes the development of promising medicines for rare conditions, for ALS. A few months later, a phase II (NCT04098406) and a multicenter phase II/III (NCT04414345) clinical trials were initiated where ALS patients were treated daily with 30 mg (or 60 mg) of CNM-Au8 for 36 and 24 weeks, respectively. The phase II clinical trial [47] was completed in 2021, and Clene Nanomoedicine announced that despite not meeting the primary and secondary biomarker endpoints (associated with preventing motor neuron loss and lung function decline), the treatment significantly slowed the disease progression and there was evidence of long-term survival benefit. This study was followed up by an optional 48-week open-label trial extension to the participants that had completed the previous study (NCT05299658). Outside of these trials, there is an expanded access program for up to 30 ALS patients to receive CNM-Au8 at the Massachusetts General Hospital (NCT04081714). Beyond ALS, an open label phase II trial (NCT03815916) in patients with Parkinson’s disease was completed in 2021 (results still pending), and there are three ongoing phase II and II/III clinical trials for patients with multiple sclerosis (NCT03536559, NCT03993171 and NCT04626921). On 15 August 2022, Clene Nanomedicine reported positive results for CNM-Au8 in the Phase II trial (NCT03536559), as the nanoformulation met the primary and secondary endpoints compared to placebo over 48 weeks. In addition, improvements across paraclinical biomarkers were reported. Despite all these completed and ongoing trials, the future of CNM-Au8 remains unclear as many of the clinical results have yet to be published.

## Conclusions

In summary, since May 2006, when CYT-6091 was first administered to patients, several therapeutic AuNP have found their way into clinical trials. These nanoconstructs share some key characteristics, which facilitate their clinical translation. In contrast to most multifunctional nanoparticles that are being reported in the literature, clinically explored AuNP tend to have simple compositions, which make their in vivo behavior easier to predict and their production easier to scale up. These nanoconstructs promote the performance of therapeutic biomolecules, such as TNF, which display higher stability and enable polyvalent interactions when conjugated on the surface of AuNP. These characteristics together with the longer circulation times upon PEG functionalization yield nanoconstructs with improved therapeutic effects that may help to decrease therapeutic doses and suppress side effects. Concerns regarding the long-term accumulation of gold in the body, however, require that the benefits of AuNP-based therapies clearly outweigh the potential risks. Examples of this include treatments for life-threatening diseases that rely on the use of gold-based nanoconstructs, such as photothermal ablation of tumors and atherosclerosis plaques with gold nanoshells, and crossing the blood–brain barrier in glioblastoma (and subsequently downregulating gene expression) with gold-based SNA. Altogether, despite not being the “magic golden bullets” initially thought, gold nanoconstructs show promising clinical results in specific niche therapies, where the unique properties of AuNP cannot be matched by other formulations.

## Data Availability

Not applicable.
